# A Novel Model for Development, Organization, and Function of Gonadotropes in Fish Pituitary

**DOI:** 10.3389/fendo.2014.00182

**Published:** 2014-10-22

**Authors:** Matan Golan, Jakob Biran, Berta Levavi-Sivan

**Affiliations:** ^1^Department of Animal Sciences, The Robert H. Smith Faculty of Agriculture, Food and Environment, The Hebrew University of Jerusalem, Rehovot, Israel

**Keywords:** zebrafish, tilapia, gonadotropin, transgene, LH, FSH

## Abstract

The gonadotropins follicle-stimulating hormone (FSH) and luteinizing hormone (LH) are key regulators of the reproductive axis in vertebrates. Despite the high popularity of zebrafish as a model organism for studying reproductive functions, to date no transgenic zebrafish with labeled gonadotropes have been introduced. Using gonadotropin regulatory elements from tilapia, we generated two transgenic zebrafish lines with labeled gonadotropes. The tilapia and zebrafish regulatory sequences were highly divergent but several conserved elements allowed the tilapia promoters to correctly drive the transgenes in zebrafish pituitaries. FSH cells reacted to stimulation with gonadotropin releasing hormone by proliferating and showing increased transgene fluorescence, whereas estrogen exposure caused a decrease in cell number and transgene fluorescence. Transgene fluorescence reflected the expression pattern of the endogenous *fshb* gene. Ontogenetic expression of the transgenes followed typical patterns, with FSH cells appearing early in development, and LH cells appearing later and increasing dramatically in number with the onset of puberty. Our transgenic lines provide a powerful tool for investigating the development, anatomy, and function of the reproductive axis in lower vertebrates.

## Introduction

The hypothalamic–pituitary–gonadal (HPG) axis is the master regulator of reproduction in vertebrates. Hypothalamic axons secrete peptides that bind to specific receptors on gonadotrope cells in the anterior pituitary and stimulate the secretion of gonadotropins (GtHs) from vesicles within these cells. The main stimulator of GtH secretion is the decapeptide gonadotropin releasing hormone (GnRH) but recently, several other hypothalamic factors have been shown to act directly on the pituitary cells to enhance GtH expression and release (1–4). In vertebrates, two GtHs have been identified: follicle-stimulating hormone (FSH) and luteinizing hormone (LH). Both GtHs are dimers comprised of a common α subunit and a distinct β subunit that confers their biological specificity. The two GtHs are produced in tetrapods by a single cell type, but play distinct roles in the regulation of gonadal processes. In the testis, FSH regulates Sertoli-cell activity and germ cell maturation whereas in females it induces germ cells and follicular growth. A LH surge is associated with ovulation in females and Leydig cell stimulation in males (5, 6).

Most experimental data in fish suggest that, as in tetrapods, FSH and LH play distinct roles in the regulation of gonadal development and function. In most species of fish, FSH is evident in the pituitary and in the plasma at early developmental stages and controls early stages of gametogenesis in both sexes. FSH is potent at inducing estrogen production by the ovary and its levels in the pituitary and plasma of females coincide with the process of vitellogenesis. LH levels are elevated during more advanced stages of gametogenesis in both males and females and induce secretion of estrogens, androgens, and maturation inducing steroids from the gonad (7, 8). In male fish, expression of the FSH receptor is not restricted to Sertoli cells and is also detected in the steroidogenic Leydig cells. This FSH-induced steroidogenesis probably regulates early stages of spermatogenesis, at a time when a LH stimulus is unavailable (9). In the ovary, both GtHs elicit estrogen production but in the mature follicles LH is more potent in inducing the production of the maturation inducing steroid 17α,20β-dihydroxy-4-pregnen-3-one (10).

A complex feedback of gonadal steroids tightly regulates GnRH receptor expression thereby affecting GtH expression and secretion. Estradiol is probably the most potent regulator of the hypothalamic–pituitary axis and generally considered to decrease *lhb* and *fshb* expression. In males, androgens play a similar role in attenuating GtH expression in the pituitary (11).

The HPG axis of fish bears striking resemblance to that of more evolved vertebrates, conserving all the major components and functions found in mammals (1, 8, 12–14). Because studies on the relationship between anatomy and function of the reproductive axis in mammals are often hindered by the inaccessibility of its components in developing and adult animals, fish models, with their unique advantages, are exceptionally valuable as a mean to enhance our understanding of the evolution of the axis and the interplay between its anatomy and function. When compared to other fish models, the zebrafish offers numerous advantages since it presents several distinct traits that make it particularly appropriate for this purpose: A large zebrafish research community has resulted in a solid knowledge base and an ever-growing array of zebrafish-related tools and resources, including methodologies, mutant lines (15), and transgenes (16). These, together with its inherent advantages such as ease of breeding and transgenesis, short generation time, transparency of embryonic stages, and so forth make zebrafish a leading choice for neuroendocrine research (13, 17).

To date several transgenic fish models with labeled GnRH neurons or gonadotrope cells have been introduced. GnRH neurons have been labeled in zebrafish (18) and medaka (19) whereas FSH was targeted in tilapia (20) and GtHs in medaka (21). In zebrafish, a line with labeled common α subunit-expressing cells was recently generated (22), but lines for identifying distinct LH and FSH producing cells have yet to be introduced.

In this study, we used regulatory elements from tilapia to drive fluorescent protein expression in zebrafish gonadotropes. Using these transgenic lines, we describe the ontogeny of GtH expression in this important model species, the anatomy of gonadotropes in the adult pituitary and demonstrate its value for testing the effects of GnRH and estrogens on GtH expression patterns.

## Materials and Methods

### Fish husbandry and breeding

Zebrafish were maintained in a stand-alone unit equipped with central filtration and heating (28 ± 1°C). The fish were fed twice daily with a commercial feed (New Life Spectrum “Grow,” New Life International Inc., Homestead, FL, USA). Breeding was performed by housing fish of both sexes in tanks with a mesh bottom. Eggs were collected in the morning and incubated until the yolk sac was completely absorbed, ca. 5 days postfertilization (dpf). Larvae were then transferred to brackish (6 ppt) water in stand-alone tanks and fed with rotifers (*Brachionus plicatilis*) until 10 dpf (23). At this age, larvae were transferred to fresh water and fed brine shrimp nauplii and dry, prepared diets. Treatment with 1-phenyl 2-thiourea was omitted in all experiments, because we observed developmental setbacks when using this treatment and because imaging quality was unaffected by the pigments. Under these conditions, fish usually reach sexual maturity at around 60 days of age.

All experimental procedures were in compliance with the Animal Care and Use Guidelines of the Hebrew University and were approved by the local Administrative Panel on Laboratory Animal Care.

### Constructs and transgenesis

The construct used for the labeling of FSH gonadotropes was as described previously (20). For the generation of labeled LH gonadotropes, we cloned a 3.6-kb fragment from tilapia genomic DNA (24) that includes a 3-kb fragment upstream of the first exon, first exon, and first intron (accession number KM575842). The primers (forward: 5′-GGGGACAA CTTTGTATAGAAAAGTTGGGCACTGAAGAAAAACGGTCCTTAA-3′; reverse: 5′-GGGGACTGCTTTTTTGTACAAACTTGGTCTGTAGGCGGCAAGTTGGA TTAGT-3′) included the appropriate attB4 and attB1R adaptors. The fragment was introduced into the pDestTol2CG2 destination clone through a LR Threeway Multisite Gateway reaction (Invitrogen, Carlsbad, CA, USA). The resulting construct drove mCherry expression in LH gonadotropes and EGFP expression in the heart. All Gateway methods and protocols were performed according to the Invitrogen Multisite Gateway Manual. For the purposes of transgenesis, eggs were collected immediately after spawning and injected with a combination of expression plasmid and transposase mRNA (16). After hatching embryos were screened for a signal in the heart and only positive embryos were grown and mated as possible founders. Tilapia transgenesis was performed as described previously (20).

### Promoter analysis

Upstream sequences of tilapia and zebrafish GtH genes were extracted from published databases (UCSC blat, https://genome.ucsc.edu). *In silico* analysis of the *cis* elements in the tilapia and zebrafish GtH promoters was performed using the Genomatix Software Suite.

### *In situ* hybridization, immunofluorescence, and imaging

To confirm correct expression of the transgene, fluorescent signals were compared to the *in situ* hybridization (ISH) staining pattern. ISH was generally performed as described previously (2–4). To detect the GtH mRNA, we cloned a fragment of the zebrafish GtH β subunit using the primers described by Ref. (25). The amplicon was cloned into the TOPO cloning vector (Invitrogen) and used as a template for the preparation of a specific digoxigenin (DIG)-labeled riboprobe (RNA DIG labeling kit, Roche Diagnostics GmbH, Mannheim, Germany). Adult fish heads were fixed overnight in 4% paraformaldehyde (PFA) at 4°C and then decalcified in 0.5 M EDTA at 4°C for 5 days. After cryoprotection [30% sucrose (w/v) in PBS overnight at 4°C] tissues were embedded in tissue freezing medium (Triangle Biomedical Sciences, Inc., Durham, NC, USA), flash frozen in liquid N_2_ and cryosectioned to 12 μm. Following ISH, the hybridization product was visualized using a fluorescent substrate (Fast Red, Roche). After confirmation of the hybridization signals, immunofluorescence (IF) labeling was performed against EGFP as previously detailed (20). Following staining, sections were mounted in anti-fade solution [2% propyl gallate (w/v), 75% glycerol (v/v) in PBS] and imaged using standard fluorescent microscopy. Since reliable mCherry antibodies were not available, the transgenic signal for this line was imaged before the ISH process. For validation of the correct expression of the LH:mCherry construct in tilapia, we applied IF using specific antibodies raised against tilapia GtH β-subunits (26, 27) and compared the two staining patterns. Sections were imaged using standard or confocal fluorescent microscopy.

For the ontogeny study, transgenic zebrafish from both lines were collected throughout their development from 4 to 65 dpf. Fish were fixed in 4% PFA overnight at 4°C. Young fish, until the age of 21 dpf were transparent enough to allow imaging of the pituitary in intact animals. For this purpose, fixed fish were cleared in 75% glycerol (v/v in PBS), mounted in anti-fade solution, and imaged ventrally on a confocal microscope. Older fish were processed for cryosectioning as described earlier and imaged using standard fluorescent microscopy.

### Real-time PCR

To assess the relative abundance of mRNAs, we used real-time PCR methodology. The genes were normalized to the amount of endogenous reference *ef1a* by the comparative threshold cycle method. Details of the method can be found elsewhere (28, 29).

### *In vivo* experiments

Larvae at the age of 10 dpf (*n* = 20) were placed in 50 ml water in petri dishes, containing the following treatments: control (10 μl ethanol in 50 ml), 100 or 1000 nM salmon GnRH analog (D-Ala6, Pro9-Net)-mammalian GnRH; sGnRHa; (Bachem, Inc., Torrance, CA, USA), 0.5, 5, or 50 ng/ml 17β-estradiol (E2; Sigma Ness Ziona, Israel), or 5 or 50 ng/ml of the aromatase inhibitor fadrozole (Novartis, Basel, Switzerland). In later experiments only a single dose of E2 (5 ng/ml) was used since the two other doses tested gave a similar response. Larvae were maintained in the treated water for 72 h. Every 24 h, all of the water in each dish was changed and treatments were added accordingly. Due to the transparency of zebrafish juveniles, we were able to image whole pituitaries in intact fish. For that purpose, at the end of the experimental period, larvae were fixed in 4% PFA and imaged ventrally using confocal microscopy. For gene-expression assays, total RNA was extracted from larvae heads using Trizol, reverse-transcribed into cDNA, as described previously (2, 29) and subjected to real-time PCR analysis. All experiments were repeated three times and a representative experiment is shown.

### Statistical analysis

Data are presented as mean ± SEM. Data were subjected to one-way ANOVA and Tukey analysis using GraphPad Prism 4 software (San Diego, CA, USA). Means marked with different letters differ significantly (*P* < 0.05).

## Results

### Gene structure and promoter comparison

**Figure 1:**
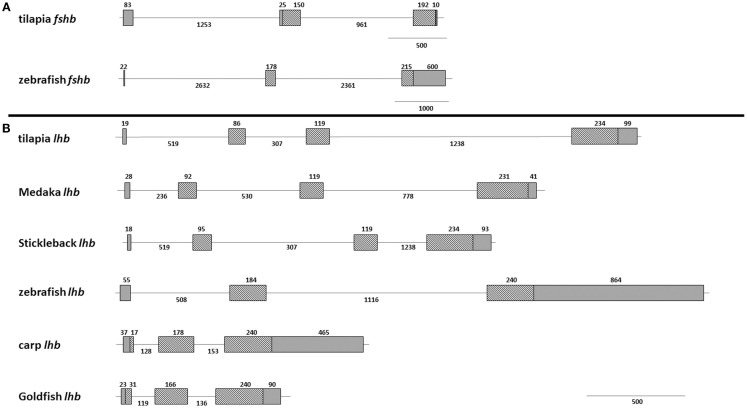
**Gene structure of fish gonadotropins**. Schematic overview of the exon–intron organization of several fish GtH genes. **(A)** The *fshb* genes of tilapia (AY294015.1 and JX887154.1) and zebrafish (NM_205624) are both divided into three exons but vary in intron size and length of untranslated regions. **(B)** The *lhb* genes of tilapia (XM_003438349.1) and zebrafish (NM_205622.2) differ in size and in exon–intron numbers. For comparison, medaka (EU_0477621) and stickleback (AJ_534969) genes contain four exons whereas carp (X_59889.1) and goldfish (30) genes are comprised of three exons. Grey boxes, untranslated regions; Hatched boxes, coding regions; lines, introns. Numbers indicate length of specified region in nucleotides.

Since we chose to use tilapia promoters to drive fluorescent protein expression in zebrafish gonadotropes, we compared the structure and composition of the GtH genes between the two species. The FSHβ gene in both species is comprised of three exons (Figure 1A). Despite retention of this basic division, the varying sizes of the exons and introns result in a more compact gene in tilapia (~2.6 kb) than in zebrafish (~6 kb). This stems from elongation of the zebrafish introns as well as a long (~600 bp) 3′ untranslated region (UTR) situated within the third exon of the zebrafish gene. In both species, the first exon is relatively short and contains only the 5′ UTR. The structure of the *lhb* gene of zebrafish varies from that of tilapia in its exon/intron division: whereas the cyprinid genes (zebrafish carp and goldfish) are comprised of three exons, in tilapia the gene is divided into four segments. Four exons are also found in the genes of medaka and stickleback (Figure 1B). The coding region size is relatively conserved at ~140 amino acids. The zebrafish *lhb* gene contains a large 3′ UTR that is twice the length of the coding region.

**Figure 2:**
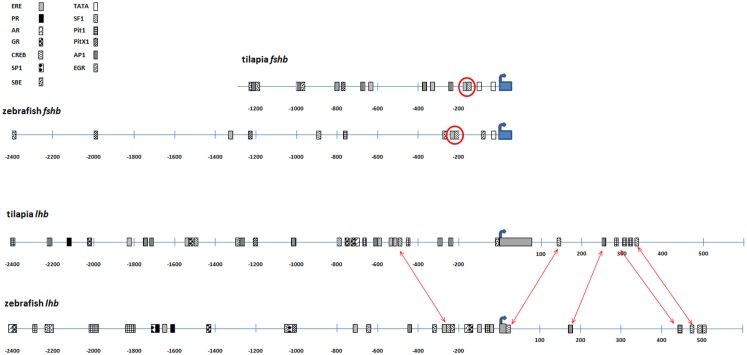
**Comparison of regulatory elements of tilapia and zebrafish gonadotropin genes**. The 5′ upstream sequence of tilapia and zebrafish GtHs was analyzed for identification of transcription factor-binding (TFB) sites. Upper panel: TFB map of the *fshb* promoters of tilapia and zebrafish. The common ERE/SF1 site is circled. Lower panel: TFB map of the *lhb* promoters of tilapia and zebrafish. This analysis also includes the first intron since common TFB sites were identified in the first introns of both species. Common TFB sites are indicated by arrows. Thick blue arrow indicates transcription start site. Gray box, first exon; ERE, estrogen-responsive element; PR, progesterone receptor-binding site; AR, androgen receptor-binding site; GR, glucocorticoid receptor-binding site; SBE, smad-binding element; EGR, early growth response-binding site.

The promoters of both *fshb* genes contain a TATA box 25 bp upstream of the transcription start site, although that of tilapia is a non-canonical variant that corresponds to the consensus sequence YYANWY (31). The tilapia promoter contains another consensus TATA sequence, at nucleotide position -95. The conserved smad- and GnRH-responsive steroidogenic factor 1 (SF1) element identified in goldfish (32) is also found in both tilapia and zebrafish proximal promoters and is adjacent to an estrogen-responsive site. Several other smad-binding sites and estrogen-responsive elements (EREs) can be found on both promoters. Pituitary-specific Pitx1 and Pit1 boxes can also be found on both promoters ~700 bp upstream of the transcription start site (Figure 2).

The combination of adjacent SF1 and ERE boxes also appeared in both proximal *lhb* promoters. Early growth response elements were also found in the zebrafish and tilapia *lhb* promoters although their position was rather distal (−650 in zf and −1300 in tilapia). Both proximal *lhb* promoters also contained the pituitary-specific Pit1 element, as well as activator protein 1 (AP1)-binding sites and numerous EREs (Figure 2). Other steroid-responsive sites included androgen and glucocorticoid receptor-binding elements. These steroid-responsive elements were also present in the more distal part of both promoters (−1000 bp and upstream). Trials to label LH gonadotropes with tilapia constructs lacking the region upstream of position-600 failed, although stable genome integration was achieved (data not shown). This implies that some of the elements found in this distal region are mandatory for correct expression of the *lhb* subunit.

We also analyzed transcription factor-binding (TFB) sites in the first intron since they were found to be important in the regulation of gene expression (33) and were thus included in our constructs. The first introns of the *fshb* genes did not exhibit significant similarity in composition and order of the TFB sites, but in the *lhb* genes, both tilapia and zebrafish first introns showed similar composition and order of several TFB sites, including cAMP-responsive elements, AP1 and Pit1 sites (Figure 2).

### Transgenes validation

**Figure 3:**
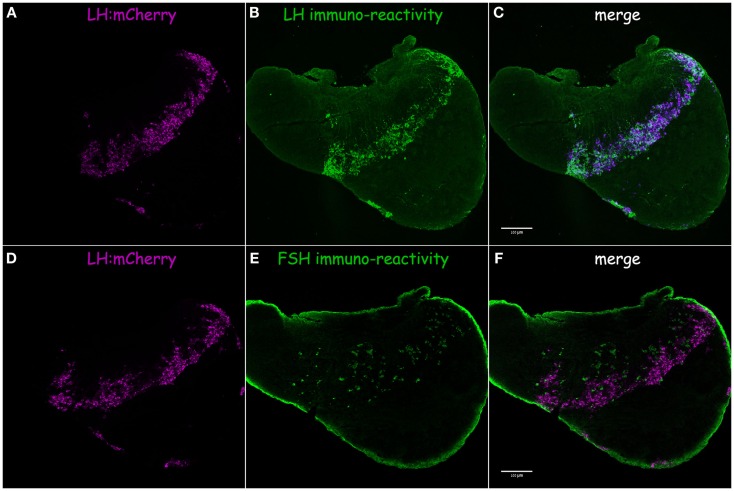
**LH:mCherry transgene validation in tilapia**. Correct expression of the fluorescent proteins in gonadotrope cells was verified by comparing the fluorescent protein expression pattern (magenta) to immunofluorescence staining using specific antibodies raised against tilapia GtH β-subunit (green). **(A)** LH:mCherry in the adult pituitary. **(B)** LHβ immunoreactivity. **(C)** Merge of A and B shows high level of colocalization of both signals. **(D)** LH:mCherry in the adult pituitary. **(E)** FSHβ immunoreactivity. **(F)** Merge of D and E shows FSH is expressed in a different subset of cells.

**Figure 4:**
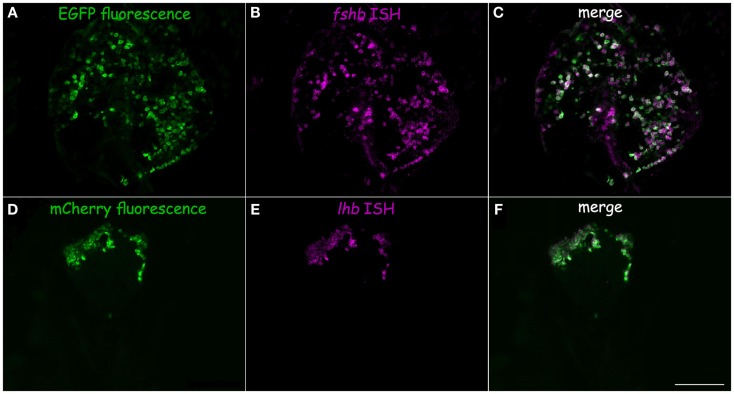
**Transgene validation in zebrafish**. Correct expression of the fluorescent proteins in gonadotrope cells was verified by comparing the fluorescent protein expression pattern (green) to fluorescent *in situ* hybridization using probes for GtH β-subunit mRNA (magenta). **(A–C)** FSH:EGFP line, **(D–F)**: LH:mCherry line. **(A)** FSH:EGFP in the adult pituitary. **(B)** *fshb* mRNA expression. **(C)** Merge of A and B shows high level of colocalization of both signals. **(D)** LH:mCherry in the adult pituitary. **(E)** *lhb* mRNA expression. **(F)** Merge of **(D)** and **(E)** shows high level of colocalization of both signals. Scale – 100 μm.

In tilapia, transgenic LH:mCherry individuals showed a high level of transgene expression in LH cells as evidenced by the colocalization of the mCherry signal in cells immunopositive for LHβ (Figures 3A–C). FSHβ immunoreactive cells represented a different subpopulation and did not express mCherry (Figures 3D–F). Both FSH:EGFP and LH:mCherry transgenic lines exhibited strong and clear expression of the fluorescent reporter in the zebrafish pituitary. Correct expression of the transgene in zebrafish was validated by comparing the expression patterns of the fluorescent reporter and that of GtH as revealed by ISH of the specific GtHβ-subunits (Figure 4). For the FSH:EGFP line, 94% (266 of 283) of the cells showed colocalization of the reporter signal and GtH expression, 3.5% (10 of 283) of the cells showed a GFP signal but no *fshb* expression (ISH), and 2.5% (7 of 283) of the cells showed *fshb* expression but no GFP signal detected by IF against GFP (Figures 4A–C). In the LH:mCherry line, the tight clustering of the cells, the unavailability of good antibodies against mCherry and the fact that the reporter tended to form aggregates inside the cells (34) made it difficult to determine the exact degree of overlap between the LHβ subunit and reporter expression. However, comparing the expression patterns of the two showed excellent colocalization of both signals (Figures 4D–F).

**Figure 5:**
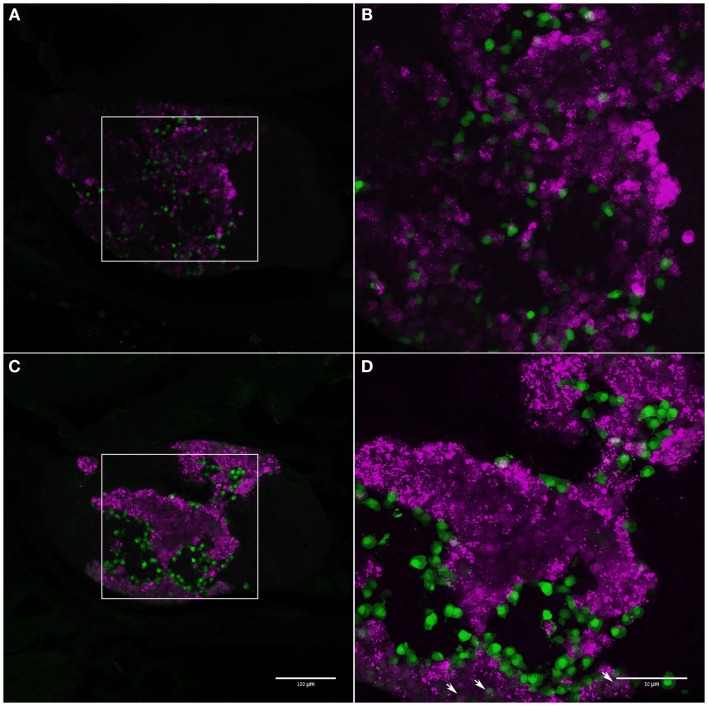
**Distribution of LH and FSH gonadotropes in the pituitary of adult zebrafish**. Adult male **(A,B)** and female **(C,D)** double-transgenic zebrafish expressing EGFP (green) in FSH gonadotropes and mCherry (magenta) in LH cells. Boxed areas in **(A)** and **(C)** mark the areas imaged in **(B,D)**, respectively (anterior – left; dorsal – up). In both sexes, FSH cells are situated at the periphery of LH-cell clumps. Strongly labeled cells do not show colocalization of both signals. Some cells in the ventral part of the pituitary cells show low expression of both signals (arrows). Scale bars for **(A,B)** are the same as for **(C,D)**, respectively.

In double-labeled zebrafish carrying both transgenes, the two cell types were easily identified and all strongly labeled cells exhibited only one type of reporter (Figure 5). However, in the ventral part of the gland, a population of cells with low-level of expression of both reporters could be identified (Figure 5D). These cells comprised <1.5% of the gonadotropes in the adult pituitary. No differences in expression patterns were found between adult males and females (Figure 5).

Contrary to a previous report in medaka (35) identifying significant extra-pituitary expression of the LH transgene, we found the expression of our LH transgene to be highly specific and restricted only to the pituitary.

### FSH transgene response to GnRH and estradiol

**Figure 6:**
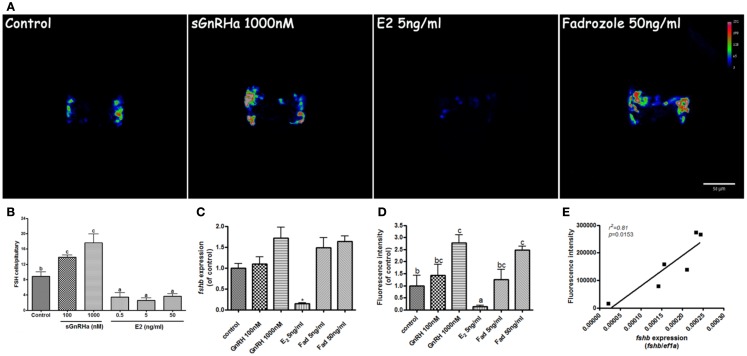
**Effect of GnRH and estrogen on *fshb* and transgene expression**. FSH:EGFP larvae at 10 days postfertilization were reared for 72 h in water containing sGnRHa (100 and 1000 nM), 17β-estradiol (E_2_, 0.5, 5, and 50 ng/ml) or the aromatase inhibitor fadrozole (5 and 50 ng/ml). **(A)** Representative heat map of the pituitary of treated fish at the end of the treatment (anterior – up). Color coding corresponds to EGFP signal intensity. GnRH and fadrozole treatment caused an increase in cell number and signal intensity. Estradiol decreased both parameters. **(B)** Quantification of the treatments effects on number of labeled cells per pituitary. GnRH increased and E_2_ decreased the number of fluorescent cells per pituitary. **(C)** Real-time quantification of the treatment effect on *fshb* gene expression also shows a significant decrease in the estradiol treatment. **(D)** Quantification of the treatment effect on fluorescent signal intensity shows an increase at the high doses of GnRH and fadrozole and a significant decrease in the estradiol treatment. **(E)** Correlation analysis of *fshb* expression and EGFP fluorescent signal intensity.

Application of GnRH in the rearing water caused an increase in FSH cell number when larvae were exposed to concentrations of 100 and 1000 nM sGnRHa from 10 to 13 dpf. In control fish, 8.9 ± 1.12 cells were found in the pituitary at this stage whereas in fish treated with GnRH, the number of cells increased to 13.9 ± 0.5 and 17.6 ± 1.7 cells in the 100 and 1000 nM treatments, respectively. Exposure to estradiol caused a marked decrease in the number of FSH cells, from the basal levels of 8.9 to 3.4 ± 1.2, 2.6 ± 0.7, and 3.7 ± 0.6 cells per pituitary in the 0.5, 5, and 50 ng/ml treatments, respectively (Figure 6B, *n* = 8). Total fluorescence in the pituitary, composed of number of fluorescent cells as well as GFP intensity, was increased by GnRH (1.7 ± 0.35 and 2.4 ± 0.48 times the control for the 100 and 1000 nM GnRH treatments, respectively; *n* = 8) and aromatase inhibitor (1.3 ± 0.39 and 2.5 ± 0.15 times basal with 5 and 50 ng/ml fadrozole, respectively; *n* = 8), and dramatically reduced by exposure to estradiol (0.1 ± 0.04 relative to basal; *n* = 8) (Figures 6A,D). To determine whether these changes also reflected the actual levels of *fshb*, we measured the expression of *fshb* by real-time PCR. The *fshb* expression levels exhibited a pattern similar to that of the fluorescence intensity in the different treatments (1.1 ± 0.3, 1.7 ± 0.4, 0.14 ± 0.02, 1.5 ± 0.3, 1.6 ± 0.2 relative to control in the control, GnRH 100, GnRH 1000, E2 5, fadrozole 5, and fadrozole 50 treatments, respectively; *n* = 8; Figure 6C). A high correlation (*r*^2^ = 0.81; *P* = 0.015) was found between *fshb* expression and gonadotrope fluorescence intensity (Figure 6E). This correlation proves that the tilapia promoter not only correctly labels zebrafish FSH gonadotropes, but also conveys physiologically relevant signals to the transgene, thereby affecting its expression levels in the same way as these signals affect the endogenous gene.

### Ontogenic expression of the transgene

**Figure 7:**
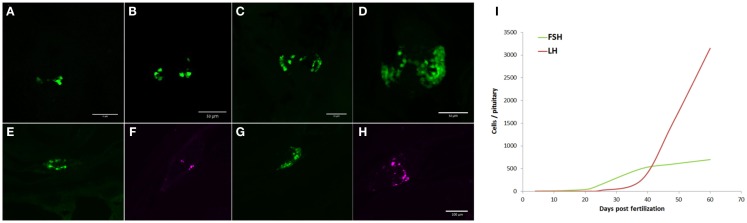
**Ontogeny of gonadotrope cells in zebrafish**. FSH (green) and LH (magenta) cell patterns were followed throughout development. **(A–D)** Proliferation of FSH cells during early development. At 4 dpf **(A)** 5–6 cells can be found in the pituitary. Cell number rises gradually as the fish develops [**(B)**-7 dpf; **(C)**-14 dpf; and **(D)**-21 dpf]. LH cells begin appearing at ~25 dpf **(F)** and increase in number toward puberty **(H)**. FSH cells continue to proliferate with age [**(E)**-28 dpf and **(G)**-38 dpf]. Images **(A–D)** are ventral whole-mount z-stacks (anterior – up). Images **(E–H)** are 12-μm thick sagittal sections [anterior – left, common scale bar in **(H)**]. **(I)** Summary of gonadotrope population dynamics from 4 dpf to adulthood.

In the present study, FSH cells appeared as early as 4 dpf, when 5–6 cells per pituitary can be detected (Figure 7A). The number of FSH cells increased with age (Figure 7) to approximately 700 cells per pituitary in the adult. LH cells were completely absent during the early life stages and only appeared (8–10 cells) at around 28 dpf (Figure 7F), their number gradually increasing thereafter (Figure 7H). At sexual maturity (~60 dpf), LH gonadotropes outnumbered FSH cells by a factor of 4.5:1 (Figures 5 and 7I). This shift in gonadotrope population implies that FSH has an important role in the developmental stages of the reproductive system whereas the role of LH becomes dominant as puberty approaches, coinciding with the transition of the ovary from primary growth to vitellogenesis in females and spermatogenesis in males.

## Discussion

In the current study, we introduce three new transgenic fish lines with fluorescently-labeled gonadotropes. The transgenic LH:mCherry tilapia joins our previously reported FSH:EGFP line (20) and completes the task of labeling gonadotropes in this emerging model species. The two zebrafish transgenic lines that were generated further enhance the value of this popular model for the study of reproductive biology.

Although the zebrafish and tilapia lineages are separated by over 250 million years of evolution (36), we could still use the promoter from tilapia to accurately drive reporter proteins in zebrafish gonadotropes. Comparison of the gene structures showed relatively high conservation in the *fshb* gene, but *lhb* subunits differed between the two species – the tilapia gene was comprised of four exons whereas the zebrafish had only three. The fourth exon can also be found in other advanced teleosts such as the medaka and stickleback. The zebrafish as well as salmon, carp, and goldfish (37) gene’s division into three exons is more reminiscent of the mammalian organization. Whereas the exon–intron division is a relatively well-conserved feature of closely related genes, addition or deletion of introns occurs at a frequency of ca. 1 intron/gene every 100 million years (38–40), and such events are common in many fish (41, 42). The fact that much of the other observed differences in mRNA size could be attributed to changes in the length of non-coding sequences, and not changes within the open reading frame, may also serve to explain the high tolerance to these significant insertions/deletions since these regions are less subject to functional constraints.

A low-level of conservation was even more salient in the gene promoters. An examination of the regulatory elements of both GtHs in tilapia and zebrafish showed very little resemblance in nucleotide sequence, and in the composition and order of the major TFB sites. Yet, the tilapia promoters drove correct reporter expression in zebrafish, suggesting that they contain enough conserved elements to enable the transgene to recruit the cell machinery in a gonadotrope-specific manner. This plasticity of the regulatory regions allows significant DNA-sequence divergence without loss of functionality (43). Our analysis showed that in the *fshb* promoters, a functional smad-response element identified in goldfish (32) also exists in both tilapia and zebrafish proximal promoters. In the distal promoters, adjacent AP1 and smad-binding element sites were also apparent in both species and found to be functional in goldfish (32). Apart from these sites, several estrogen-receptor- and SF-binding sites were present in the regulatory regions. The presence of these sites can explain the high responsiveness of the transgene to estrogen exposure. A strong response of *fshb* expression to estrogen exposure has also been observed in salmon (44) and mammals (45). Since GnRH is the key stimulator of *fshb* expression and proliferation (28, 46), it is not surprising that exposure of larvae to high doses of GnRH caused an increase in transgene and *fshb* expression. Because an *in vivo* model was used in this study, it is difficult to determine whether estrogen and GnRH exerted their effects directly, by binding to their cognate receptors on the gonadotrope, or indirectly by paracrine or autocrine effects – by affecting other regulatory pathways, which control gonadotrope proliferation and expression. Nevertheless, the fact that our transgene can react to external stimuli in a physiologically relevant context strengthens its value and reliability. Moreover, this model can be easily applied to test for the presence and physiological effect of estrogenic substances simply by exposing transgenic fish to potentially contaminated water and then directly quantifying fluorescence intensity of the gonadotropes.

The regulatory region of the *lhb* gene also showed little similarity in nucleotide sequence and transcription factor organization between tilapia and zebrafish. However, an ERE/SF1 complex that was found in the *fshb* promoters was also found in both *lhb* sequences. This combination plays an important role in inducing *lhb* transcription (47). Early growth response-binding elements are also vital for *lhb* gene expression (37) and can be found on both promoters in an intermediate position (tilapia at −1332; zebrafish at −707). A PitX1-binding site in the proximal promoter was found to be fundamental for the activation of the *lhb* gene in chinook salmon (48) and play an important role in mammalian *lhb* gene regulation (49). The additional Pitx1-binding site is present in the tilapia proximal *lhb* promoter, close to the ERE/SF1 element, but in the zebrafish, it is located further upstream at −1032. Many other EREs are located in the proximal and distal promoters of tilapia and zebrafish and may account for the reported effects of estrogens on *lhb* expression. In general, in most fish species examined estrogens had an overall increasing effect on *lhb* gene expression and secretion whereas the effect on FSH was less consistent (7). In sub-adult salmon, estrogens had a profound up-regulating effect on *lhb* and a downregulating effect on *fshb* gene expression (44). In tilapia, estradiol has been shown to decrease *fshb* and *lhb* expression *in vivo* and decrease effect of GnRH on GtH secretion (28). In primary cell cultures from the adult zebrafish pituitary, estrogen increased expression of both GtHs (50). Gonadectomy studies further support the notion of tight feedback mechanisms exerted by gonadal steroids on GtH expression and secretion levels. When gonads are removed pituitaries reacted to the decrease in gonadal steroid levels by increasing GtH production, dependent on the reproductive stage of the fish (51–53).

Introns are incorporated into many transgenic cassettes as a mean of increasing transgene expression in mammals (54–56), fish (57, 58), and invertebrates (59). The effect of introns on transgene performance can be attributed to the fact that the 5′ region (first 500–1000 nt from the splice site) of the first intron in many genes exhibits an exceptional degree of conservation and has been shown to contain functional regulatory elements (33). Since we included the first intron of both genes in our constructs, we also tested for similarities within these sequences. In the *lhb* introns, a specific sequence of putative binding motifs was found in both zebrafish and tilapia, although the motifs were more densely distributed in the tilapia, corresponding to the trend toward a more compact genome in tilapia than in zebrafish (42).

Our model shows good separation between the expression patterns of the two gonadotropes, evidenced by the fact that all clearly labeled cells expressed only one GtH, in both zebrafish and tilapia. However, very few cells in the ventral-most part of the proximal pars distalis showed low expression levels of both fluorescent proteins. While fish are generally considered to have complete separation of the two GtH types (8), there is some evidence of coexpression of both hormones within the same cells in the Mediterranean yellowtail (60). Another explanation relies on the concept that both LH and FSH are derived from the same lineage (61), and the fact that FSH gonadotropes begin appearing in this area and gradually migrate to their final, more dorsal position (25), thus marking the ventral pituitary as the gonadotrope’s “birthplace.” It is possible that these ventral cells represent a portion of the population that is going through phases of differentiation and may experience a bipotent stage before committing to their final roles.

The developmental pattern of FSH and LH found in our fish corresponds well with that described by Chen and Ge (25) for the same species. FSH was expressed at relatively early stages of development whereas the appearance of LH cells was highly correlated with the onset of puberty, as is the case for tilapia and mummichog (62, 63). This large gap in the temporal expression of FSH and LH differs from the situation in mammals, in which both GtHs are expressed at approximately the same time during development (64). The early expression of FSH may be related to the fact that at these initial stages, FSH seems to play developmental roles in processes other than reproduction, and depletion of its β-subunit mRNA causes severe developmental abnormalities (25). In accordance with the concept of a non-reproductive role for early FSH expression, we could not find any GnRH3 fibers reaching the pituitary until 7 dpf, and even at 14 dpf, some of the fish failed to exhibit GnRH axons in the pituitary (data not shown), although a significant number of FSH cells were present in the pituitary at that time. This observation is strengthened by the fact that in mammals, FSH cells are unresponsive to GnRH at early developmental stages and require LH signaling to begin expressing GnRH receptor (65). This activation by LH is apparently not necessary in fish, as in our model at 14 dpf the FSH cells were receptive to GnRH stimuli, as evidenced by the increase in *fshb* expression in response to GnRH application (despite a lack of LH at this stage).

Our transgenic zebrafish lines have been validated using accepted methodologies and the transgene response to estrogens and GnRH – the key modulators of GtH secretion – reflects the endogenous gene response patterns. Moreover, the transgene ontogeny profiles closely follow those described for this species. Nevertheless, it is important to note that the tilapia regulatory elements, operating within the zebrafish genomic environment, may respond differently from the endogenous zebrafish genes to some relevant stimuli. Factor-specific validation is therefore required when applying this model to investigate other aspects of GtH regulation.

In summary, we used regulatory elements from an evolutionary distant species to drive correct expression of transgenes in zebrafish gonadotropes. Our results highlight the functional conservation of highly diverged genomic regulatory regions. Our newly introduced transgenic zebrafish lines provide an important and powerful tool for investigating the differential development, anatomy, and function of the reproductive axis in vertebrates.

## Conflict of Interest Statement

The authors declare that the research was conducted in the absence of any commercial or financial relationships that could be construed as a potential conflict of interest.
